# Localization of Arabidopsis FORKED1 to a RABA-positive compartment suggests a role in secretion

**DOI:** 10.1093/jxb/erx180

**Published:** 2017-06-01

**Authors:** Neema Prabhakaran Mariyamma, Hongwei Hou, Francine M Carland, Timothy Nelson, Elizabeth A Schultz

**Affiliations:** 1 Department of Biological Sciences, University of Lethbridge, Lethbridge, AB, TIK, Canada; 2 Department of Molecular, Cellular and Developmental Biology, Yale University, New Haven, CT, USA

**Keywords:** FORKED1, leaf vein patterning, PIN1 localization, RABA, SCARFACE/VAN3, secretory pathway, vascular differentiation

## Abstract

When *FORKED1* (*FKD1*) is mutated, asymmetric localization of PINFORMED1 (PIN1), particularly to the apical side of cells, fails to occur properly in developing veins, resulting in an open vein pattern. *FKD1* encodes a protein with a Pleckstrin homology-like (PL) domain, suggesting interaction with phosphoinositides. FKD1 has been previously found to interact with an ADP ribosylation factor GTPase-activating protein (ARF-GAP) important for vein patterning, SCARFACE/VAN3 (SFC). We find that FKD1–green fluorescent protein (GFP) localizes to the plasma membrane and to punctae labeled by SFC–yellow fluorescent protein (YFP). Supporting the idea that the FKD1 PL domain recognizes phosphatidylinositol 4-phosphate [PtdIns(4)P], FKD1–GFP co-localizes with PtdIns(4)P markers, and is more cytosolic when in a background mutant for the PtdIns(4,5)P_2_ hydrolases CVP2 and CVL1. Both FKD1 and SFC partially co-localize with markers for the *trans*-Golgi network (TGN), at which endocytic and secretory pathways merge. FKD1-labeled punctae rarely co-localize with the endocytic marker FM4-64, suggesting that FKD1 is not involved primarily in the endocytic pathway. FKD1 and SFC co-localize with members of the RABA group of RAB-GTPases, which are proposed to act in the post-Golgi secretory pathway. The compartments labeled by FKD1 and SFC do not localize to membrane compartments induced by the fungal toxin brefeldin A (BFA). Collectively, our data suggest that FKD1 and SFC act in a BFA-insensitive secretory pathway.

## Teaser

FORKED1 is required for proper PIN1 localization within developing veins. Our findings that FORKED1 localizes to the plasma membrane, *trans*-Golgi network, and RABA-positive compartments suggest a role in secretion.

## Introduction

Leaves are the primary organs involved in the perception of light and conversion of solar energy into organic carbon. The leaf veins provide both mechanical support and transport routes for photosynthetic substrates and products. Underscoring their importance, changes in leaf vein pattern through evolutionary time are correlated with more efficient leaf performance (Boyce *et al.*, 2009; Brodribb and Feild, 2010; Zwieniecki and Boyce, 2014). Whereas primitive plants such as ferns, progymnosperms, and gymnosperms typically have an open venation pattern characterized by bifurcating veins (Roth-Nebelsick *et al.*, 2001), eudicots exhibit a characteristic hierarchical network pattern, with small veins emerging from larger veins to form a closed reticulum (Nelson and Dengler, 1997). The evolution of multiple vein orders is thought to improve both structural support and physiological performance (Zwieniecki *et al.*, 2002; Feugier *et al.*, 2005; Brodribb *et al.*, 2016).

The formation of the hierarchal, reticulate vein pattern typical of angiosperms is proposed to be driven by directed transport and canalization of the hormone auxin (Sachs, 1981). In molecular terms, the direction and canalization of auxin flux is controlled by cellular expression of the auxin efflux protein PINFORMED1 (PIN1), its polar localization, and its dynamic relocalization, which in turn enhances auxin flux (Scarpella *et al.*, 2006; Wenzel *et al.*, 2007; Bayer *et al.*, 2009). During formation of second-order veins, PIN1 expression domains in the ground tissue, associated with high levels of auxin in the epidermis, extend and eventually connect to the PIN1 expression domain of the midvein. These early second-order PIN1 expression domains, referred to as the lower loop domains (LLDs) are composed of a transient wide distal section near the epidermis and a persistent narrow proximal section near the midvein (Scarpella *et al.*, 2006; Wenzel *et al.*, 2007). PIN1 localization is dynamic within cells of the LLD, beginning as symmetric or lateral and becoming increasingly basal. The upper loop domain (ULD) gradually extends from the LLD and establishes two segments with opposite PIN1 polarity bridged by a single bipolar cell. Cells in the segment adjacent to the LLD have basal PIN1 (towards the LLD) and cells in the segment adjacent to the midvein have apical polarity (towards the midvein). Higher order vein formation reiterates this process (Scarpella *et al.*, 2006; Wenzel *et al.*, 2007). Thus, the formation of the reticulate vein pattern requires apical localization of PIN1 within those cells of the newly forming vein that connect distally to the previously formed vein.

Localization of PIN proteins involves a complex interplay between endocytic, recycling, and secretory pathways, which have been dissected, in part, through analysis of the involvement of various ADP ribosylation factor guanine nucleotide exchange factors (ARF-GEFs). The ARF-GEF GNOM regulates recycling of endocytosed PIN1 to the basal cell membrane (Geldner *et al.*, 2003; Naramoto *et al.*, 2011) and acts redundantly with GNOM-LIKE1 (GNL1) to control early secretory events targeting newly synthesized PIN1 to the basal membrane (Doyle *et al.*, 2015). ARF-GEFs BIG1–BIG4 act at the *trans*-Golgi network (TGN) to target newly synthesized PIN1 to the plasma membrane in a non-polar fashion (Richter *et al.*, 2014). The targeting of PIN1 to the basal membrane is sensitive to the fungal toxin brefeldin A (BFA), due to the involvement of the BFA-sensitive GNOM. However, targeting of PIN1 to the apical membrane, which does not normally occur within the root or stem, can be induced by prolonged BFA exposure, implying that this pathway is insensitive to BFA (Kleine-Vehn *et al.*, 2008*a*).

Mutations to a number of genes alter the dynamics of PIN1 localization and canalization within the leaf procambium, and result in altered vein reticulation. SCARFACE (SFC; also known as Vascular Network Defective 3-VAN3) is an ARF-GTPase-activating protein (GAP) protein belonging to the class 1 (AGD3) of AGD proteins (Koizumi *et al.*, 2005; Sieburth *et al.*, 2006). In *sfc/van3* mutants, PIN1 polarity is established but fails to be maintained in pre-procambial cells (Scarpella *et al.*, 2006) resulting in fragments of vascular tissue in place of continuous secondary and higher order veins. Weak alleles of GNOM result in a phenotype opposite to *sfc/van3*, producing cotyledons with more veins and increased connections. Double mutants result in mutual suppression of both phenotypes, suggesting that SFC/VAN3 and GNOM might be acting in opposing pathways (Sieburth *et al.*, 2006), an idea supported by the co-localization of GNOM and SFC/VAN3 at the plasma membrane (Naramoto *et al.*, 2011). Moreover, the co-localization of GNOM and SFC/VAN3 with clathrin led to the proposal that SFC/VAN3 may be involved in clathrin-mediated endocytosis (Naramoto *et al.*, 2011).

SFC/VAN3 contains a Plekstrin homology (PH) domain that is often associated with phosphoinositide binding, and, indeed, SFC/VAN3 binds with high affinity to phosphatidylinositol 4-phosphate [PtdIns(4)P] (Koizumi *et al.*, 2005). SFC/VAN3 co-localizes with SYP41, suggesting its localization to the TGN (Koizumi *et al.*, 2005), a localization that requires the wild-type PH domain (Naramoto *et al.*, 2009). Underscoring the importance of phosphoinositides to vein formation, mutation to *COTYLEDON VASCULAR PATTERN2* (*CVP2*) or *CVP2 LIKE1* (*CVL1*) genes, which encode type I inositol polyphosphate 5-phosphatases 6 (At5PTase6) and generate PtdIns(4)P from PtdIns(4,5)P_2_, phenocopies the *sfc/van3* phenotype (Carland and Nelson, 2009). In *cvp2cvl1* mutants, SFC/VAN3 localization becomes cytosolic, supporting the idea that recruitment of SFC/VAN3 to the TGN requires interaction with PtdIns(4)P probably through the PH domain (Carland and Nelson, 2009; Naramoto *et al.*, 2009).

Mutations to *FORKED1* (*FKD1*) (also known as *VAN3 BINDING PROTEIN, VAB*; Naramoto *et al.*, 2009) result in a failure to establish consistent basal localization of PIN1–green fluorescent protein (GFP) within pre-procambial cells, a failure to establish apical localization of PIN1–GFP within the bipolar cell and the ULD, and a subsequent reduction in vein connections (Hou *et al.*, 2010). Double mutants between *fkd1* and *sfc/van3* are more severe than the single mutants, suggesting that the genes may act together (Steynen and Schultz, 2003), a proposal supported by the finding that the two interact in yeast two-hybrid assays and in bimolecular fluorescence complementation (Naramoto *et al.*, 2009). FKD1 contains a Pleckstrin-like (PL) domain, and either it or the SFC/VAN3 PH domain is sufficient for localization of both proteins (Naramoto *et al.*, 2009). The formation of a FKD1–SFC complex, and the defective PIN1–GFP localization in *sfc/van3* and *fkd1* mutants, suggests that FKD1 and SFC may be involved in establishing appropriate PIN1 localization in provascular cell files, thereby regulating vascular differentiation.

Beyond its association with SFC/VAN3, the molecular function of FKD1 is not well characterized. To understand the precise subcellular localization of FKD1 and potential interactors in vesicle trafficking pathways, we performed co-localization of FKD1 with markers for different endomembrane compartments. In this work, we establish that FKD1, together with SFC, localizes to a BFA-insensitive RABA-positive compartment that partially overlaps with TGN markers and that FKD1 also localizes to the plasma membrane. We propose that FKD1 and SFC act in a post-Golgi secretory pathway between the TGN and plasma membrane, and that this pathway is important for PIN1 targeting to the apical membrane in provascular cells.

## Materials and methods

### Seed and DNA stocks

As described previously, (Pahari *et al.*, 2014), *Arabidopsis thaliana* was sown on soil or plates with AT growth medium (Ruegger *et al.*, 1998), left at 4 °C for 3 d, and transferred to a growth chamber. The date of transfer was considered 0 days after germination (DAG). *Nicotiana tabacum* and *Nicotiana benthamiana* were grown on soil. The PIPlines (Simon *et al.*, 2014) UBQ10:CITRINE–2×FYVE(HRS), UBQ10:CITRINE–2×PH(FAPP1), and UBQ10:CITRINE–2×PH(PLCD1), and the Wave lines (Geldner *et al.*, 2009) Wave 13Y [UBQ10:YFP (yellow fluorescent protein)–VTI12], Wave34Y (UBQ10:YFP–RabA1e), Wave2Y (UBQ10:YFP–RabF2b/ARA7), Wave131Y (UBQ10:YFP–NPSN12), and Wave138Y (UBQ10:YFP–PIP1;4) were obtained from the Arabidopsis Biological Resource Centre (ABRC) at Ohio State University, USA. Syp61pro:syp61–CFP (cyan fluorescent protein) seeds (Robert *et al.*, 2008) were donated by Dr Marisa Otegui, University of Wisconsin, USA. 35S:GFP–RABA1c seeds (Qi and Zheng, 2013) were donated by Dr Hugo Zheng, McGill University, Canada. *pi4kβ1β2* seeds (Preuss *et al.*, 2006) were obtained from Dr Erik Nielsen, University of Michigan, USA. The Columbia (Col-0) ecotype was used as a wild-type control in all experiments.

Vectors containing full-length cDNA for FKD1 (U16276), members of the ARF gene family (U09053, ARF1A1a-At1g23490; U21558, ARF1A1c-At2g47170; U09461, ARFA1d-At1g70490; and U12397, ARFA1e-At3g62290) and the pnigel 7 (UBQ10:YFP) vector (Geldner *et al.*, 2009) were obtained from the ABRC. pVKH18–GFPC (Batoko *et al.*, 2000) and 35S:GFP–RABA1c (Qi and Zheng, 2013) were obtained from Hugo Zheng (McGill University, QC, Canada), 35S:RFP–RABA1b (Choi *et al.*, 2013) from Dr. Takashi Ueda, University of Tokyo, Japan, and 35S:YFP–RABA4b (Preuss *et al.*, 2004) from Dr Erik Nielsen, University of Michigan, USA. 35S:SYP61–YFP (Stefano *et al.*, 2010) and 35S:ST–RFP (red fluorescent protein) (Wee *et al.*, 1998) were obtained from Dr Federica Brandizzi, Michigan State University, USA.

### Generation of transgenes for transient or stable expression

To generate the 35S:FKD1–GFP construct, FKD1 cDNA was amplified from U16276 using primers BAM1FKDcDNA (GGA TCCATGGAGAGAGAACTCAGACG) and FKDcDNASalI (GGTCGACTTAAGGTGCCATCCATTTG), and ligated into binary vector pVKH18-GFPC at *Bam*HI and *Sal*I restriction sites. To isolate SFC cDNA, RNA was isolated from 7 DAG Col-0 seedlings using the TRIzol extraction method (Ambion), treated with DNase (Turbo; Applied Biosystems), and further purified with RNeasy MinElute Cleanup (Qiagen). cDNA was generated using Retroscript primed with oligo(dT) for first-strand synthesis according to the manufacturer’s instructions (Amersham). Primers SFC-7 (GCTGTTTGTGTCGAAGAAGAG) and SFC-9 (CCGCAAGCCTATGCATCT) were used to amplify SFC cDNA which was subsequently cloned into the pCR2.1 TOPO vector (Invitrogen), sequenced to confirm fidelity, and recombined into the pEARLEYGATE101 vector (Earley *et al.*, 2006) using LR Clonase (Invitrogen Cat. No. 11791020), according to the manufacturer’s instructions. Integration of ARF1 genes into UBQ10::YFP was done by recombining their cDNAs with the pnigel7 vector and the products sequenced as described in Geldner *et al.*, 2009. Transgenes 35S:FKD1–GFP and 35S:SFC–YFP were transformed via *Agrobacterium tumefaciens* into Arabidopsis lines *fkd1* and *fkd2-1*, an allele of SFC (Steynen and Schultz, 2003; Carland and Nelson, 2009), using the floral spray method. Transformed plants were selected for hygromycin resistance (35S:FKD1–GFP) or Basta resistance (35S:SFC–YFP). Several independent lines were screened for vein phenotype, transgene expression and protein localization, and a representative line chosen.

### Protein co-localization by stable or transient expression

To analyze the co-localization of FKD1 or SFC with other proteins, stable transgenic lines were made by crossing plants homozygous for 35S:FKD1–GFP with plants homozygous for UBQ10:YFP–VTI12, UBQ10:YFP–RabA1e, UBQ10:YFP–RaF2b/ARA7, UBQ10:YFP–NPSN12, UBQ10:YFP–PIP1;4, UBQ10:CITRINE–2×FYVE(HRS), UBQ10:CITRINE–2×PH(FAPP1), UBQ10:CITRINE–2×PH(PLCD1), or 35S:SFC–YFP, or by crossing plants homozygous for 35S:SFC–YFP with plants homozygous for Syp61pro:syp61:CFP or 35S: GFP–RABA1c. The localization of 35S:FKD1–GFP with the organelle marker UBQ10:YFP–VTI12 was done in the F_1_ generation. Homozygous lines of 35S:FKD1–GFP with UBQ10:YFP–NPSN12, UBQ10:YFP–PIP1;4, UBQ10:YFP–RabF2b/ARA7, UBQ10:YFP–RabA1e, UBQ10:CITRINE–2×FYVE(HRS), UBQ10:CITRINE–2×PH(FAPP1), UBQ10:CITRINE–2×PH(PLCD1), or 35S:SFC –YFP, and 35S:SFC–YFP with 35S:GFP–RABA1c or SYP61Pro: SYP61–CFP were identified in the F_3_ or subsequent generation based on resistance to hygromycin (35S:FKD1–GFP and 35S:GFP–RABA1c), Basta [35S:SFC–YFP, UBQ10:YFP–NPSN12, UBQ10:YFP–PIP1;4, UBQ10:YFP–RabF2b/ARA7, UBQ10:YFP–RabA1e, UBQ10:CITRINE–2×FYVE(HRS), UBQ10:CITRINE–2×PH(FAPP1), UBQ10:CITRINE–2×PH(PLCD1)] or kanamycin (SYP61Pro:Syp61–CFP). For transient expression in *N. tabacum* or *N. benthamiana*, the abaxial leaf epidermis was injected with *Agrobacteriu*m strains harboring appropriate binary vectors following a previous protocol (Batoko *et al.*, 2000).

### Introduction of FKD1–GFP into mutant lines

In order to analyze the localization of FKD1 in mutants defective in phosphoinositide production, a line homozygous for 35S:FKD1–GFP was crossed to *cvp2cvl1* and *pi4kβ1β2*, and the F_1_ was backcrossed to the double mutant. Homozygous plants for 35S:FKD1–GFP with *cvp2cvl1* and 35S:FKD1–GFP with *pi4kβ1β2* were obtained in the F_3_ generation by screening for the double mutant phenotype, resistance to hygromycin (35S:FKD1–GFP), and fluorescence by confocal microscopy. Because *cvp2cvl1* develops slowly, we assessed characteristics of 2.5 DAG cotyledon pavement cells in the 35S:FKD1–GFP line and in *cvp2cvl1* expressing 35S:FKD1–GFP. Cell perimeter (P) and area (A) were measured using NIH image on cells viewed using differential interference contrast on a Olympus Fluoview FV1000 confocal microscope, and the undulation index (UI) was calculated using the formula UI=P/[2π√(A/π)] (Minamisawa *et al.*, 2011). Means were compared for statistical differences by the Student’s *t*-test. The localization of 35S:FKD1–GFP was assessed in the epidermal pavement cells of cotyledon cells at 2.5 DAG for all genotypes. An ANOVA was carried out using the PROC MIXED procedure of SAS (SAS Institute), followed by a Fisher’s test to determine statistical differences between means.

### Confocal imaging and analysis

*Nicotiana tabacum* or *N. benthamiana* leaves 48 h post-injection or Arabidopsis roots or cotyledons at 2.5 DAG were mounted in water and viewed using a Olympus Fluoview FV1000 confocal microscope. For BFA treatment, 2.5 DAG seedlings were incubated in 50 μM BFA for 3 h before viewing the seedlings; control seedlings were incubated in solution without BFA, but with DMSO at the same concentration as the BFA-treated seedlings. For FM4-64 treatment, 2.5 DAG seedlings were incubated in 16 mM FM4-64 for 15 min, rinsed in water, and then viewed after a further 15, 30, and 45 min. For plasmolysis experiments, 2.5 DAG seedlings were incubated in 800 mM mannitol for 3 h followed by a 2 min water rinse. For comparison of 35S:FKD1–GFP localization at different stages of development, GFP was excited with laser 473 nm (emission filters 485–585 nm). For co-localization experiments involving GFP or YFP and RFP/FM4-64, GFP/YFP and RFP/FM4-64 were excited with a 473 nm laser (emission filters 485–545 nm) and a 559 nm laser (emission filters 570–670 nm), respectively. For co-localization experiments involving YFP and CFP, YFP and CFP were excited sequentially with 405 nm and 515 nm lasers, respectively. For co-localization experiments involving GFP with YFP, GFP was excited with a 458 nm laser (emission filters 470-496 nm) and YFP with a 515 nm laser (emission filters 530–600 nm). Imaging was carried out using the line-sequential scanning mode, and all images used in comparisons were taken at the same confocal settings. To assess the co-localization of proteins, Pearson’s coefficient of correlation (PCC) values were obtained within single cells (cotyledons and leaves) or files of cells (roots) using the PSC co-localization plugin in NIH Image J software (developed at the US National Institutes of Health and available at http://rsb.info.nih.gov/nih-image/). For all co-localization analyses, at least 15 samples were analyzed for each experiment. Images shown in the figures are representative of average co-localization patterns for the samples whenever possible. Images were processed with Adobe Photoshop Elements version 5.0 software (Adobe Systems).

## Results

### FKD1 localization is developmentally regulated and localization is influenced by phosphoinositides

**Fig. 1.**
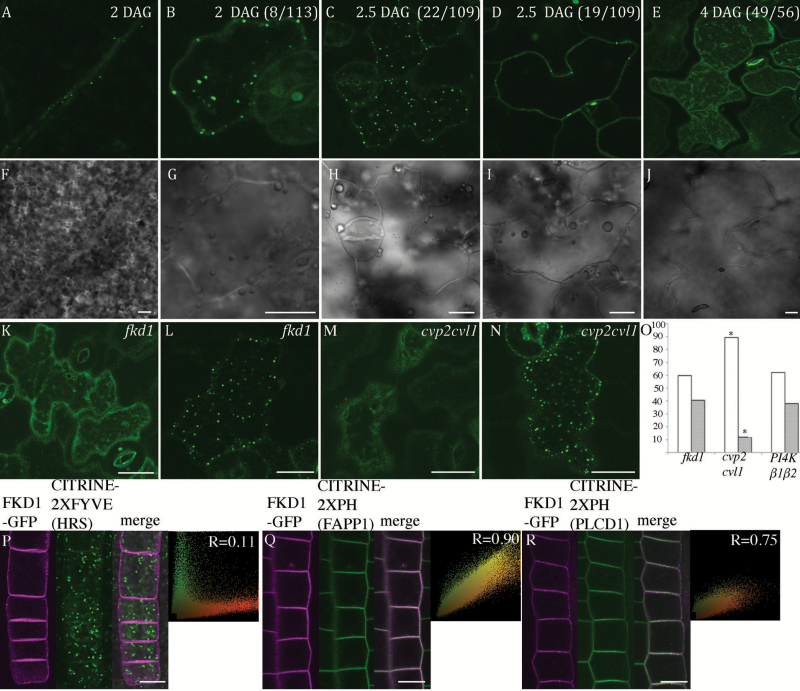
35S:FKD1–GFP localization is developmentally regulated and correlated with the phosphoinositide profile. 35S:FKD1–GFP localization in 2 DAG vascular cells (A) and in epidermal pavement cells at sequential days of cotyledon development (B, 2 DAG; C and D, 2.5 DAG; and E, 4 DAG). Corresponding bright field images are shown below (F–I). Comparison of 35S:FKD1–GFP localization in cotyledon pavement cells of *fkd1* and *cvp2cvl1* mutant plants. Localization in the cytosol and punctae in *fkd1* (K and L, respectively) and in *cvp2cvl1* mutant plants (M and N, respectively). (O) Graph representing the percentage of cotyledon pavement cells with 35S:FKD1–GFP localization in the cytosol (open bars) and punctae (gray bars) in *fkd1* (*n*=30), *cvp2cvl1* (*n*=33) and *PI4Kβ1β2* (*n*=15) mutants. An asterisk indicates significantly different from the wild type (Fisher’s test, *P*<0.05). Co-localization of 35S:FKD1–GFP with markers for the phosphoinosities PtdIns(3)P (P), PtdIns(4)P (Q), and PI4,5P (R) in root epidermal cells. Within (P), (Q), and (R), sections are as labeled [FKD1–GFP alone, PtdIns(3)P marker UBQ10:CITRINE–2×FYVE(HRS), PtdIns(4)P marker UBQ10:CITRINE–2×PH(FAPP1), or PtdIns(4,5)P_2_ marker UBQ10:CITRINE–2×PH(PLCD1), the merged images, and the scatter plots of the corresponding merged image with Pearson’s coefficient of correlation (*R*) values]. Scale bar=10 μm).

*FKD1* is essential for the auxin response that directs vascular differentiation in developing cotyledons and leaves (Steynen and Schultz, 2003), and is important in localizing PIN1–GFP within developing leaf vein cells (Hou *et al.*, 2010). Introduction of 35S:FKD1–GFP into *fkd1* mutants results in a wild-type leaf vein phenotype (see Supplementary Fig. S1C at *JXB* online), suggesting that transcription driven by the 35S promoter provides adequate spatial and temporal expression, and the fusion protein is functional. In 2 DAG cotyledons, punctae of 35S:FKD1–GFP are visible in developing vascular cells (Fig. 1A), but expression is too weak to provide sufficient samples for reliable comparison. Thus, cotyledon epidermal cells were used for further analysis. We noticed that 35S:FKD1–GFP localization in cotyledon epidermal cells is different in cotyledons at different ages. At 2 DAG, when pavement cells are more or less circular in shape (see brightfield image Fig. 1G), 35S:FKD1–GFP is cytosolic in most cells (73%, *n*=113 cells), but is associated with the plasma membrane and/or in punctae in ~27% of cells (Fig. 1B). At 2.5 DAG, when cells are expanding and initiating lobes and indentations (Fig. 1H), more cells have 35S:FKD1–GFP associated with punctae (Fig. 1C; 20%, *n*=109 cells) or with punctae and the plasma membrane (Fig. 1D; 17%, *n*=109 cells). Lobe initiation is complete by 3 DAG (Zhang *et al.*, 2011), and 35S:FKD1–GFP localization is again more cytosolic at both 3 DAG (79%, *n*=81 cells) and 4 DAG (Fig. 1E; 88%, *n*=56 cells). Because 2.5 DAG pavement cells have the highest association of 35S:FKD1–GFP with punctae and the plasma membrane, all subsequent analyses were done at this stage.

VAN3/SFC and FKD1 form a complex which has been proposed, through their respective PH or PL domains, to bind to PtdIns(4)P, a product generated by inositol polyphosphate 5' phosphatases (5PTases) such as CVP2 and CVL1 (Carland and Nelson, 2004, 2009). When both *CVP2* and *CVL1* are mutated, VAN3 is mislocalized from the TGN to the cytosol (Naramoto *et al.*, 2009). To investigate whether 35S:FKD1–GFP localization is similarly altered in *cvp2cvl1* mutants, 35S:FKD1–GFP was introduced into the *cvp2cvl1* mutant background. *cvp2cvl1* mutants develop more slowly than the wild type (Carland and Nelson, 2009). To determine if cotyledon epidermal cells of 2.5 DAG *cvp2cvl1* mutants expressing FKD1–GFP were at a similar stage to those in the FKD1–GFP line, we measured area (A) and perimeter (P), then calculated the UI according to the formula UI=P/[2π√(A/π)] (Minamisawa *et al.*, 2011). We found no significant differences in these characters (Supplementary Table S1), suggesting that epidermal pavement cells in the two genotypes are at a similar stage of development at 2.5 DAG. Observation of 35S:FKD1–GFP in cotyledons of both genotypes at 2.5 DAG indicates that 35S:FKD1–GFP localization is significantly more cytosolic in *cvp2cvl1* mutants (89%, *n*=507 cells) than in *fkd1* mutants (63%, *n*=232 cells) (Fig. 1K–O).

The phospholipid signaling pathway of Arabidopsis includes three families of phosphoinositide kinases, of which PI4Ks can yield PI4(P). In Arabidopsis, two PI4KIIIβ (PI4KIIIβ1 and PI4KIIIβ2) genes have been identified (Mueller-Roeber, 2002). We asked whether FKD1 localization and expression is altered in plants mutant for both genes (Preuss *et al.*, 2004). 35S:FKD1–GFP was introduced into *PI4Kβ1β2* mutants, and 2.5 DAG cotyledons were analyzed for 35S:FKD1–GFP localization. No difference was observed in 35S:FKD1–GFP localization in *PI4Kβ1β2* mutants compared with the original 35S:FKD1–GFP line (Fig. 1O).

To assess which phosphoinositides FKD1 associates with *in vivo*, we created lines expressing 35S:FKD1–GFP and markers for different phosphoinositides (PIPlines; Simon *et al.*, 2014): PtdIns(3)P [UBQ10:CITRINE–2×FYVE(HRS)], PtdIns(4)P [UBQ10:CITRINE–2×PH(FAPP1)], or PtdIns(4,5)P_2_ [UBQ10:CITRINE–2×PH(PLCD1)]. The PIPlines are not all expressed in cotyledon pavement epidermal cells, thus we focused on co-localization within the root protoderm (Fig. 1P–R). In root protoderm cells, as in cotyledon pavement cells, FKD1–GFP is localized to the plasma membrane and to punctae in some cells, and to the cytosol in other cells. While localization to the punctae was visible when FKD1–GFP was excited with the 473 nm laser (Fig. 5; Supplementary Fig. S2A), only plasma membrane localization was visible when FKD1–GFP was excited with the 458 nm laser with a reduced emission spectrum (470–496 nm), as when co-localization with YFP/CITRINE was carried out (Fig. 1P–R). FKD1–GFP did not co-localize with the PtdIns(3)P marker UBQ10:CITRINE–2×FYVE(HRS) (PCC=0.07 ± 0.08, *n*=29 cell files; Fig. 1P), but co-localized very strongly with the PtdIns(4)P marker UBQ10:CITRINE–2×PH(FAPP1) (PCC=0.91 ± 0.04, *n*=22 cell files; Fig. 1Q) and co-localized moderately with the PtdIns(4,5)P_2_ marker UBQ10:CITRINE–2×PH(PLCD1) (PCC=0.69 ± 0.16, *n*=25 cell files; Fig. 1R). The co-localization of FKD1–GFP with PtdIns(4)P and PtdIns(4,5)P_2_, both of which localize to the plasma membrane (Simon *et al.*, 2014), indicates that in root protodermal cells, FKD1 is strongly associated with the plasma membrane.

### FKD1 co-localizes with plasma membrane and post-Golgi markers

To establish that FKD1 is localized at the plasma membrane and to understand the nature of the punctae, we determined the co-localization of FKD1 with a set of markers specific for the plant endomembrane system in both stable (Arabidopsis cotyledon epidermal cells) and transient expression (*N. tabaccum* or *N. benthamiana* leaf epidermal cells) systems.

**Fig. 2.**
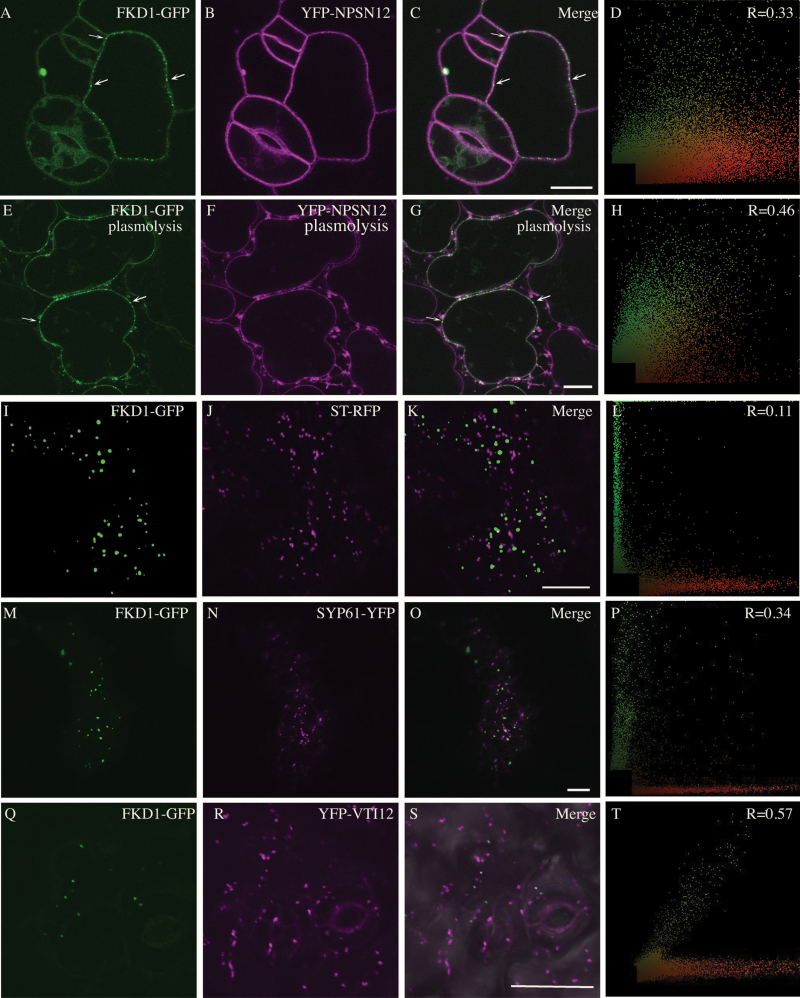
35S:FKD1–GFP localizes to the plasma membrane and *trans*-Golgi, but not to the Golgi. Localization in stably transformed 2.5 DAG Arabidopsis cotyledon pavement cells of 35S:FKD1–GFP with the plasma membrane marker UBQ10:YFP–NPSN12 (A–H) before (A–D) and after (E–H) plasmolysis treatment (800 mM mannitol, 3 h). (A and E) 35S:FKD1–GFP alone; (B and F), UBQ10:YFP–NPSN12; (C and G) merged images; (D and L) scatter plots of the merged image with Pearson’s coefficient of correlation (*R*) values. Arrows in (A), (C), (E), and (G) indicate bright foci of FKD1–GFP localization. Localization in transiently expressing *Nicotiana tabacum* of 35S:FKD1–GFP with the Golgi marker 35S:ST–RFP (I–L) or TGN marker 35S:SYP61–YFP (M–P); (I and M) 35S:FKD1–GFP alone; (J) 35S:ST–RFP; (N) 35S:SYP61–YFP. (K and O) Merged images; (L and P) scatter plots of the merged image with Pearson’s coefficient of correlation (*R*) values. Localization in stably transformed 2.5 DAG Arabidopsis cotyledon pavement cells of 35S:FKD1–GFP with the TGN marker UBQ10:YFP–VTI12 (Q–T); (Q) 35S:FKD1–GFP alone, (R) UBQ10:YFP–VTI12, (S) merged images, (T) scatter plot of the merged image with Pearson’s coefficient of correlation (*R*) value. Scale bar=10 μm.

In cotyledon epidermal cells, 35S:FKD1–GFP associated moderately with both plasma membrane markers: UBQ10:YFP–NPSN12 (average PCC 0.33 ± 0.14, *n*=43 cells; Fig. 2A–D; Table 1) and UBQ10:YFP–PIP1;4 (average PCC 0.22 ± 0.15, *n*=32 cells; Supplementary Fig. S3A–D; Table 1). Following plasmolysis of Arabidopsis seedlings expressing 35S:FKD1–GFP and UBQ10:YFP–NPSN12 or UBQ10:YFP–PIP1;4, 35S:FKD1–GFP signal remains similarly associated with the plasma membrane label (average PCC 0.39 ± 0.15, *n*=28 cells and 0.32 ± 0.15, *n*=38 cells, respectively), providing further evidence that FKD1 is associated with the plasma membrane (Fig. 2E–H; Supplementary Fig. S3E–H; Table 1). Interestingly, whereas a proportion of FKD1–GFP is, like UBQ10:YFP–NPSN12 and UBQ10:YFP–PIP1;4, uniformly distributed throughout the membrane, FKD1–GFP is also localized to bright, plasma membrane-associated foci (arrows, Fig. 2A, C, E, G).

We next set out to better define the nature of the punctae to which 35S:FKD1–GFP localizes. In *N. tabacum* epidermal cells, as in Arabidopsis cotyledon epidermal cells, 35S:FKD1–GFP is frequently cytosolic but does show clear localization to punctae in a proportion of cells. We focused our analysis on those cells in which FKD1–GFP was localized to punctae. In these cells, 35S:FKD1–GFP does not co-localize with ST–RFP (average PCC= –0.30 ± 0.12, *n*=22 cells; Table 1; Fig. 2I–L), a marker of the Golgi apparatus (Wee *et al.*, 1998). Partial localization to the TGN/early endosome (EE) (Bassham *et al.*, 2000; Sanderfoot *et al.*, 2001; Drakakaki *et al.*, 2012) was indicated by 35S:FKD1–GFP moderately co-localizing with markers 35S:SYP61–YFP (PCC=0.37 ± 0.10, *n*=38 cells; Fig. 2M–P) in *N. tabacum* epidermal cells and UBQ10:YFP–VTI12 (PCC=0.39 ± 0.10, *n*=39 cells; Fig. 2Q–T) in Arabidopsis pavement cells. Taken together, our results suggest that FKD1 is localized to the plasma membrane, as well as to a subset of the TGN.

### FKD1 co-localizes strongly with SFC/VAN3 (ARF-GAP) and with ARF1 proteins

**Fig. 3.**
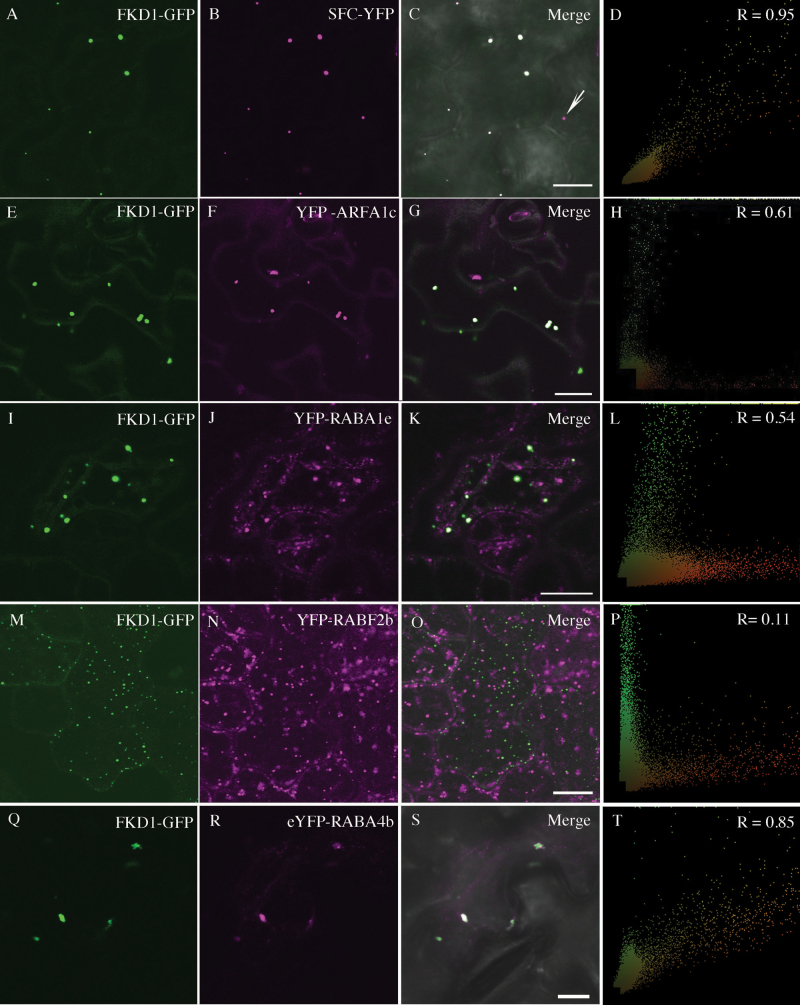
35S:FKD1–GFP localizes strongly with 35S:SFC–YFP, UBQ10:YFP–ARFA1c, and RABA–GTPases. Localization of 35S:FKD1–GFP with 35S:SFC–YFP (A–D), UBQ10:YFP–ARFA1c (E–H), UBQ10:YFP–RabA1e (I–L), UBQ10:YFP–RABF2b (M–P), or 35S:eYFP–RABA4b (Q–T) in stably transformed 2.5 DAG Arabidopsis cotyledon pavement cells (A–D and I–P) or in *Nicotiana tabacum* leaf epidermal cells (E–H and Q–T). (A), (E), (I), (M), and (Q) 35S:FKD1–GFP alone; (B) 35S:SFC–YFP alone; (F) UBQ10:YFP–ARFA1c alone; (J) UBQ10:YFP–RabA1e alone; (N) UBQ10:YFP–RABF2b alone; (R) 35S:eYFP–RABA4b alone; (C), (G), (K), (O), and (S) merged images; (D), (H), (L), (P), and (T) scatter plots of the merged image with Pearson’s coefficient of correlation (*R*) values. The arrow in (C) indicates a puncta labeled by SFC–YFP but not FKD1–GFP. Scale bar=10 μm).

**Fig. 4.**
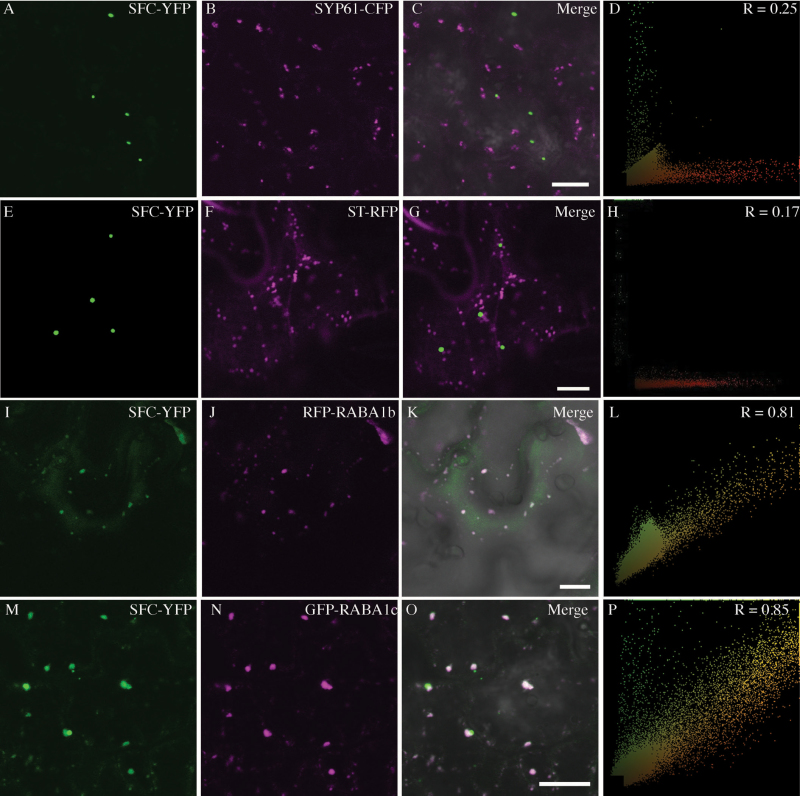
35S:SFC–YFP localizes moderately with the TGN marker SYP61pro:SYP61–CFP and strongly with RABA-GTPases. Localization of 35S:SFC–YFP with the TGN marker marker SYP61pro:SYP61–CFP (A–D), the Golgi marker 35S:ST–RFP (E–H), or secretory markers 35S:RFP–RABA1b (I–L) and 35S:GFP–RABA1c (M–P) in stably transformed 2.5 DAG Arabidopsis cotyledon pavement cells (A–D and M–P), transiently expressing *Nicotiana tabacum* leaf epidermal cells (E–H) or transiently expressing *Nicotiana benthamiana* leaf epidermal cells (I–L). (A), (E), (I), and (M) 35S:SFC–YFP alone; (B) SYP61pro:SYP61–CFP alone; (F) 35S:ST–RFP alone; (J) 35S:RFP–RABA1b alone; (N) 35S:GFP–RABA1c alone; (C), (G), (K), and (O) merged images; (D), (H), (L), and (P) scatter plots of the merged image with Pearson’s coefficient of correlation (*R*) values. Scale bar=10 μm.

Previous experiments using transient expression in a heterologous system (the leaf epidermis of *N. bethamiana*) showed that VAB–YFP (FKD1–YFP) and VAN3–GFP co-localized to dot-like structures (Naramoto *et al.*, 2009). To confirm that FKD1 and SFC co-localize in Arabidopsis and to understand further the localization of SFC, we introduced 35S:SFC–YFP into the *fkd2* mutant, an allele of *SFC* (Steynen and Schultz, 2003; Carland and Nelson, 2009). Reversion of the *fkd2* leaf vein phenotype to wild type (Supplementary Fig. S1E) indicated that the fusion protein was functional and the 35S promoter sufficient for transcription. We next created stable transgenic lines expressing 35S:FKD1–GFP and 35S:SFC–YFP, and analyzed their localization patterns. 35S:FKD1–GFP co-localizes strongly with 35S:SFC–YFP in cotyledon epidermal cells (PCC=0.90 ± 0.08, *n*=56 cells; Fig. 3A–D; Table 1) and in root cells (PCC=0.73 ± 0.15, *n*=32 cells; Supplementary Fig. S2C–F; Table 1). In cotyledon cells, FKD1–GFP-labeled punctae are almost always also labeled by SFC–YFP, whereas a subset of SFC–YFP-labeled punctae are consistently not labeled by FKD1–GFP (arrow in Fig. 3C). Interestingly, we noticed that 35S:FKD1–GFP localizes more consistently to punctae in the presence of SFC than in its absence. Whereas only 37% of 2.5 DAG cotyledon cells (*n*=109) expressing 35S:FKD1–GFP alone had FKD1–GFP localized to punctae, all of 2.5 DAG cotyledon cells (*n*=56) co-expressing 35S:FKD1–GFP and 35S:SFC–YFP have FKD1–GFP localized to punctae. Like 35S:FKD1–GFP, 35S:SFC–YFP shows moderate co-localization with SYP61Pro:SYP61–CFP (average PCC=0.22 ± 0.12, *n*=23 cells; Fig. 4A–D) and does not associate with ST–RFP (average PCC= –0.26 ± 0.14, *n*=18 cells; Fig. 4E–H).

While the ARF substrate of SFC is unknown, likely candidates are the ARFA1 proteins, which have been proposed as the potential substrates of SFC based on sequence similarity to their mammalian counterparts (Sieburth *et al.*, 2006). Six highly similar ARFA1 proteins exist in Arabidopsis (ARFA1a–ARFA1f) (Vernoud *et al.*, 2003). Cellular localization of only ARFA1c has been established, and it has been found to localize to the Golgi apparatus and TGN (Pimpl *et al.*, 2000; Xu and Scheres, 2005; Stefano *et al.*, 2006, 2010). We performed transient expression in *N. tabaccum* of 35S:FKD1–GFP with four members of the ARF gene family (ARFA1a, ARFA1c, ARFA1d, and ARFA1e) fused to YFP. Consistent with their localization to the TGN, 35S:FKD1–GFP shows strong co-localization with UBQ10:YFP–ARFA1c (average PCC=0.63 ± 0.13, *n*=23 cells; Fig. 3E–H). Supporting functional redundancy among the ARFA1 family, FKD1–GFP co-localized to a similar extent with all of UBQ10:YFP–ARFA1a, UBQ10:YFP–ARFA1d, and UBQ10:YFP-ARFA1e (average PCC=0.66 ± 0.25, *n*=34 cells; 0.76 ± 0.15, *n*=30 cells; and 0.43 ± 0.29, *n*=36 cells respectively; Supplementary Fig. S4).

### 35S:FKD1–GFP and 35S:SFC–YFP co-localize weakly with endocytic markers

The endocytic and secretory pathways merge within the TGN (Viotti *et al.*, 2010); thus, we sought to understand with which pathway FKD1 is associated. First, we determined that FKD1–GFP co-localized strongly with UBQ10:YFP–RabA1e (average PCC=0.54 ± 0.16, *n*=35 cells; Fig. 3I–L; Table 1), which is considered a marker of the early and recycling endosomes (Geldner *et al.*, 2009). In contrast, we found that FKD1 co-localizes weakly with UBQ10:YFP–RabF2b/ARA7 (PCC=0.13 ± 0.14, *n*=42 cells; Fig. 3M–P; Table 1), a marker of the late endosome/pre-vacuolar complex (Ueda *et al.*, 2004; Geldner *et al.*, 2009; Ebine *et al.*, 2011).

**Table 1.** Correlation of expression between the intensities of 35S:FKD1–GFP and 35S:SFC–YFP together with different proteins fused to YFP or RFP in root or cotyledon epidermis of stably transformed Arabidopsis seedling at 2.5 DAG or in leaf epidermis of transiently transformed *N. tabacum* or N. *benthamiana*

	Sample size (*n*)	Tissue type	Mean PCC
**Co-localization of 35S:FKD1–GFP with:**			
UBQ10:CITRINE–2×FYVE(HRS)	29	Arabidopsis root	0.07 ± 0.08
UBQ10:CITRINE–2×PH(FAPP1)	22	Arabidopsis root	0.91 ± 0.04
UBQ10:CITRINE–2×PH(PLCD1)	25	Arabidopsis root	0.69 ± 0.16
pUBQ10:YFP–NPSN12 (mannitol untreated)	43	Arabidopsis cotyledon	0.33 ± 0.14
pUBQ10:YFP–NPSN12 (mannitol treated)	28	Arabidopsis cotyledon	0.39 ± 0.15
pUBQ10:YFP–PIP1;4 (mannitol untreated)	32	Arabidopsis cotyledon	0.22 ± 0.15
pUBQ10:YFP–PIP1;4 (mannitol treated)	38	Arabidopsis cotyledon	0.32 ± 0.15
35S:ST–RFP	22	*N. tabaccum* leaf epidermis	–0.30 ± 0.12
35S:SYP61–YFP	38	*N. tabaccum* leaf epidermis	0.37 ± 0.10
pUBQ10:VTI12–YFP	39	Arabidopsis cotyledon	0.39 ± 0.20
35S:SFC–YFP	56	Arabidopsis cotyledon	0.90 ± 0.08
pUBQ10:ARFA1a–YFP	34	*N. tabaccum* leaf epidermis	0.66 ± 0.25
pUBQ10:ARFA1c–YFP	23	*N. tabaccum* leaf epidermis	0.63 ± 0.13
pUBQ10:ARFA1d–YFP	30	*N. tabaccum* leaf epidermis	0.76 ± 0.15
pUBQ10:ARFA1e–YFP	36	*N. tabaccum* leaf epidermis	0.43 ± 0.29
pUBQ10:YFP–RABA1e	35	Arabidopsis cotyledon	0.54 ± 0.16
pUBQ10:YFP–RABF2b	42	Arabidopsis cotyledon	0.13 ± 0.14
35S:eYFP–RABA4b	22	*N. tabaccum* leaf epidermis	0.65 ± 0.25
35S:SFC–YFP (BFA untreated)	32	Arabidopsis root	0.73 ± 0.15
35S:SFC–YFP (BFA treated)	38	Arabidopsis root	0.63 ± 0.14
**Co-localization of 35S:SFC–YFP with:**			
SYP61pro:SYP61–CFP	23	Arabidopsis cotyledon	0.22 ± 0.12
35S:ST–RFP	18	*N. tabaccum* leaf epidermis	–0.26 ± 0.14
RFP–RABA1b	46	*N. benthamiana* leaf epidermis	0.84 ± 0.05
35S:GFP–RABA1c	44	Arabidopsis cotyledon	0.82 ± 0.12

Sample size is the number of cells (cotyledons and leaves) or files of cells (roots).

PCC is the mean of PCC from all merged images determined using co-localization plugin in NIH Image J.

**Fig. 5.**
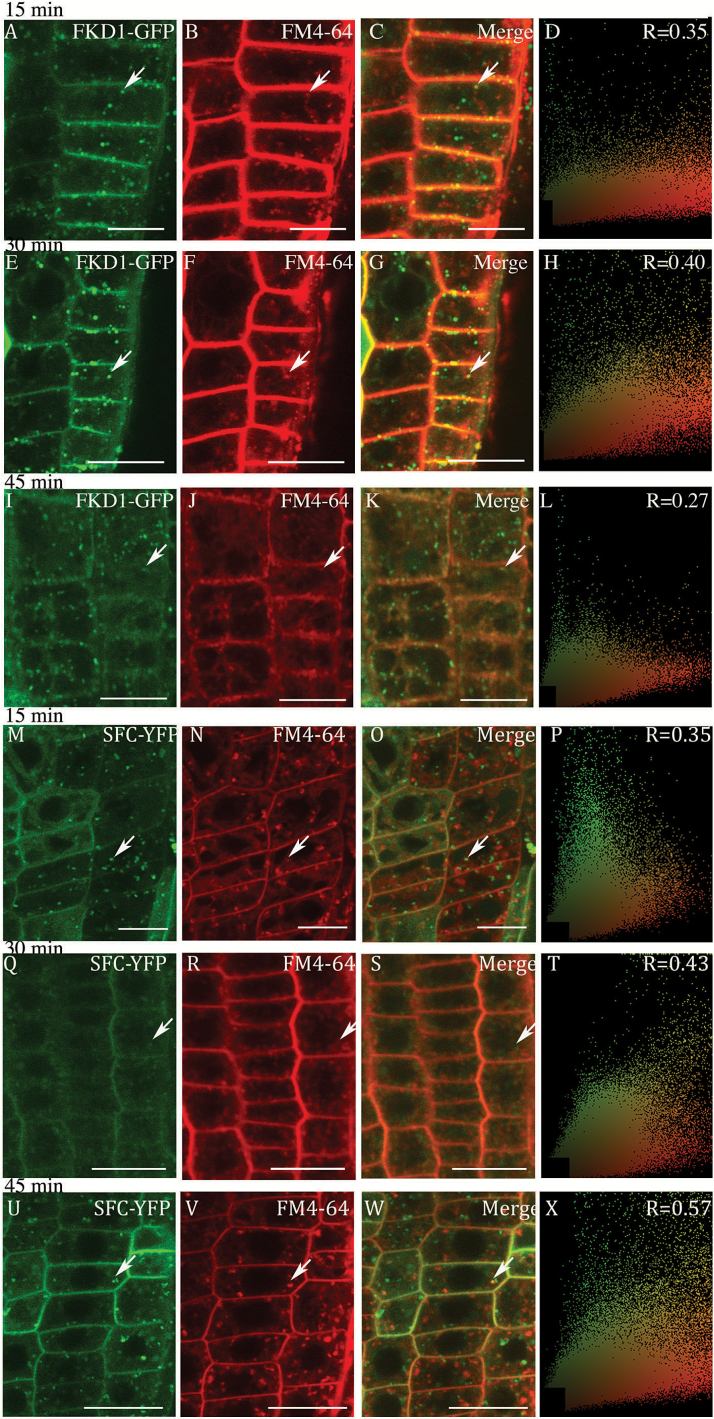
Co-localization of 35S:FKD1–GFP or 35S:SFC–YFP with FM4-64 at different time intervals. Epidermal ells in the root elongation zone expressing 35S:FKD1–GFP (A–L) or 35S:SFC–YFP (K–X) stained with the endocytic tracer FM4-64 after 15 min (A–D; M–P), 30 min (E–H; Q–T), and 45 min (I–L; U–X). (A), (E), and (I) 35S:FKD1–GFP alone; (M), (Q), and (U) 35S:SFC–YFP alone; (B), (F), (J), (N), (R), and (V) FM4-64 alone; (C), (G), (K), (O), (S), and (W) merged images; (D), (H), (L), (P), (T), and (X) scatter plots of the merged image with Pearson’s coefficient of correlation (*R*) values. Arrows indicate punctae labeled by both FKD1–GFP or SFC–YFP and FM4–64. Scale bars=10 μm.

To confirm the localization of FKD1–GFP within the EE, we treated 35S:FKD1–GFP-expressing seedlings with the fluorescent endocytic tracer FM4-64. FM4-64 labels the plasma membrane, and is taken into the cell interior by endocytosis, after which it gradually labels the entire endocytic pathway (Bolte *et al.*, 2004; Ueda *et al.*, 2004). Seedlings were treated with FM4-64 for 15 min, rinsed, and vesicles co-localizing with FM4-64 were observed after 15, 30, and 45 min. FM4-64 co-localized with 35S:FKD1–GFP at the plasma membrane, as indicated by a PCC of ~0.27 at all time points (Fig. 5A–L; Table 2). Surprisingly, when only punctae were considered, FM4-64 co-localized with very few 35S:FKD1–GFP-expressing punctae at all time points (15 min, 2%, *n*=963 punctae; 30 min, 5%, *n*=909 punctae; 45 min, 6%, *n*=966 punctae; arrows in Fig. 5A–L; Table 2). The small proportion of 35S:FKD1–GFP punctae localizing with FM4-64 suggests that FKD1 is not acting predominantly in the endocytic pathway. While our data indicate very strong co-localization of FKD1 with SFC, SFC/VAN3 has been suggested to have a role in clathrin-mediated endocytosis (Naramoto *et al.*, 2011). To explore this paradox, we assessed co-localization of FM4-64 with 35S:SFC–YFP. Association of SFC–YFP-expressing punctae with FM4-64 label was slightly higher than that of FKD1–GFP at the 15 min time point (7%, *n*=1837 punctae; Table 2; Fig. 5M–P), and increased to 21% (*n*=1711 punctae) at 30 min (Table 2; Fig. 5Q–T) and 23% (*n*=1761 punctae) at 45 min (Table 2; Fig. 5U–X). While the co-localization of SFC–YFP with FM4–64 supports the idea that a proportion of SFC/VAN3 may be acting in endocytosis, the low level of FKD1–GFP and SFC–YFP association with FM4-64 suggests that the proteins do not act primarily in the endocytic pathway.

**Table 2.** Correlation of expression between 35S:FKD1–GFP- or 35S:SFC–YFP- and FM4-64-labeled structures at different times

Correlation of marker with FM4-64	15 min	30 min	45 min
PCC 35S:FKD1–GFP	0.27 ± 0.30 (*n*=33)	0.27 ± 0.29 (*n*=38)	0.26 ± 0.30 (*n*=28)
Percentage of 35S:FKD1–GFP punctae	2% (*n*=963)	5% (*n*=909)	6% (*n*=966)
PCC 35S:SFC–YFP	0.20 ± 0.11 (*n*=42)	0.48 ± 0.23 (*n*=38)	0.50 ± 0.14 (*n*=33)
Percentage of 35S:SFC–YFP punctae	7% (*n*=1837)	21% (*n*=1711)	23% (*n*=1761)

PCC is the mean of PCC from all merged images determined using co-localization plugin in NIH Image J; *n* is the number of cell files analyzed.

Percentage is the mean percentage of punctae labeled by 35S:FKD1–GFP or 35S:SFC–YFP that is also labeled by FM4-64; *n* is the number of punctae analyzed

We asked if FKD1 and SFC might be acting in the secretory pathway by assessing their co-localization with several RAB-GTPases of the RABA group (Preuss *et al.*, 2006; Asaoka *et al.*, 2013; Qi and Zheng, 2013). In tobacco epidermal cells, 35S:FKD1–GFP co-localized strongly with 35S:eYFP–RABA4b (average PCC=0.65 ± 0.25, *n*=22 cells; Fig. 3Q–T; Table 1) which is involved in secretion in both root hairs and pollen tubes (Preuss *et al.*, 2006; Qi and Zheng, 2013). We attempted to assess co-localization of both SFC–YFP and FKD1–GFP with RFP–RABA1b in *N. tabacum*; however, in our hands, RFP–RABA1b was almost entirely cytosolic in this system. We next expressed the fusion proteins in *N. benthamiana*, as had been previously reported, and saw frequent localization of RFP–RABA1b to the punctae. In this system, FKD1–GFP was entirely cytosolic, but SFC–YFP was localized to discrete punctae that frequently co-localized with RFP–RABA1b (average PCC=0.84 ± 0.05, *n*=46 cells; Fig. 4I–L; Table 1). We also assessed co-localization of SFC–YFP with GFP–RABA1c in cotyledon pavement cells of stably transformed Arabidopsis, and found a very similar, strong co-localization (PCC=0.82 ± 0.12, *n*=44 cells; Fig. 4M–P; Table 1). The strong association of FKD1–GFP and SFC–YFP with RAB-GTPases acting in the secretory pathway, combined with their weak association with the endocytic marker FM4-64, suggests that a significant proportion of the proteins act in the secretory pathway.

### FKD1 and SFC are insensitive to BFA

As described above, FKD1 associates with SFC/VAN3, an ARF-GAP, and also with members of the ARF1 family, raising the possibility that FKD1 might associate with an ARF-GEF. Since ARF-GEFs can be categorized based on their sensitivity to the fungal toxin BFA, we sought to eliminate possible ARF-GEF associations by assessing the BFA sensitivity of FKD1 and SFC.

**Fig. 6.**
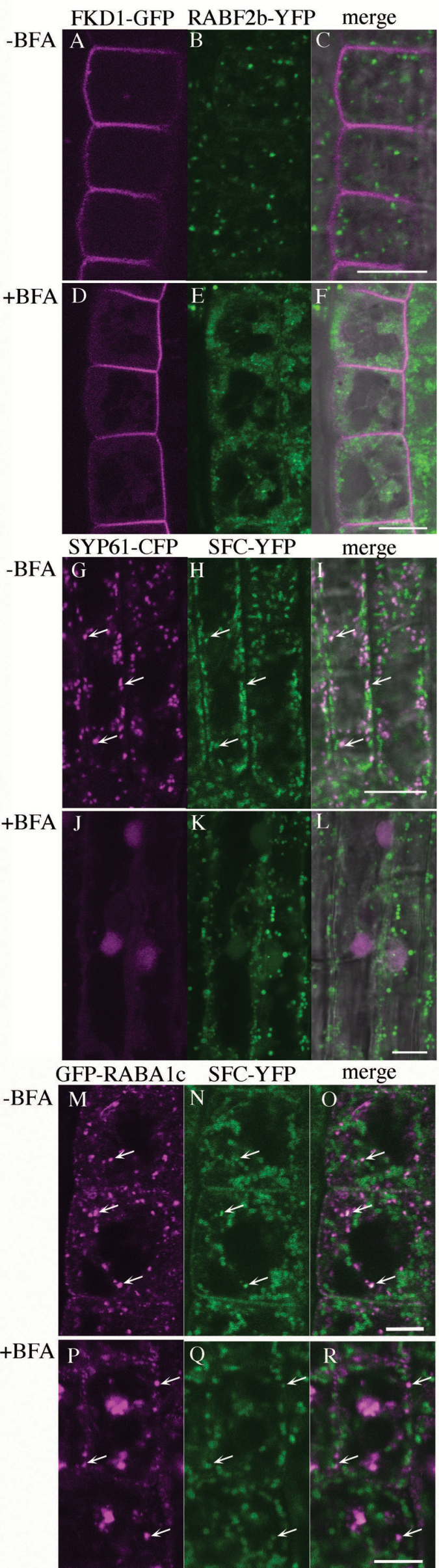
35S:FKD1–GFP and 35S:SFC–YFP compartments are unaffected by BFA treatment. Localization of 35S:FKD1–GFP with UBQ10:RABF2b–YFP (A–F), 35S:SFC–YFP with SYP61pro:SYP61–CFP (G–L), or 35S:SFC–YFP with 35S:GFP–RABA1c in Arabidopsis root epidermal cells treated with DMSO (A–C; G–I; M–O) or 50 μM BFA in DMSO (D–F; J–L, P–R). (A and D) 35S:FKD1–GFP alone; (H), (K), (N), and (O) 35S:SFC–YFP alone; (B and E) UBQ10:RABF2b–YFP alone, (G and J) SYP61pro:SYP61–CFP alone; (M and P) 35S:GFP–RABA1c alone; (C), (F), (I), (L), (O), and (R) merged images. Arrows in (G–I) and (M–R) indicate co-localization of the two labels. Scale bar=5 μm.

Comparison of 35S:FKD1–GFP localization in BFA-untreated and BFA-treated roots revealed no differences, indicating that FKD1 compartments are insensitive to BFA treatment in root tissues (compare Supplementary Fig. S2A and S2B). To compare the behavior of FKD1 compartments with BFA-induced compartments, we exposed seedlings expressing both 35S:FKD1–GFP and UBQ10:YFP–RabF2b/ARA7 to BFA. Following exposure, the UBQ10:YFP–RabF2b/ARA7 marker localized to BFA compartments in root epidermal cells, whereas 35S:FKD1–GFP remained localized to the plasma membrane and not BFA compartments (compare Fig. 6A–C with 6D–F). We next asked whether SFC was similarly insensitive to BFA by treating seedlings expressing both 35S:SFC–YFP and SYP61Pro:SYP61–CFP or 35S:GFP-RABA1c with BFA. Again, whereas SYP61Pro:SYP61–CFP and 35S:GFP–RABA1c localized to BFA compartments, 35S:SFC–YFP remained localized to small, discrete punctae in root cells (compare Fig. 6G–I with 6J–L and Fig. 6M–O with P–R). Interestingly, SYP61–CFP almost entirely localized to BFA compartments, while a proportion of GFP–RABA1c did not become incorporated into the BFA compartments. We observed a similar proportion of vesicles labeled by both SFC–YFP and GFP–RABA1c in BFA-untreated and BFA-treated root cells (arrows in Fig. 6M–R), implying that the BFA-insensitive compartment is not homogenous. To determine further whether the FKD1–SFC complex is sensitive to BFA, seedlings expressing 35S:FKD1–GFP and 35S:SFC–YFP were treated with BFA and compared with untreated seedlings. No difference was observed either in the morphology of FKD1 and SFC vesicles or in their co-localization patterns (Supplementary Fig. S2; Table 1). The insensitivity of FKD1 and SFC compartments to BFA suggests that, if they are associated with an ARF-GEF, it is likely to be a BFA-resistant member.

## Discussion

### FKD1 and SFC associate within the secretory pathway

Our data confirm that FKD1 and SFC are strongly associated, with all FKD1–GFP-labeled punctae also labeled by SFC–YFP. Moreover, we suggest that the FKD1/SFC complex is localized within a RABA-positive subcompartment of the TGN that is required for transport from the TGN to the plasma membrane. Both 35S:FKD1–GFP and 35S:SFC–YFP partially co-localize with markers of the TGN/EE (VTI12–YFP/SYP61–YFP and SYP61–CFP, respectively), and strongly co-localize with members of the RABA group (RABA4b and RABA1b/RABA1c, respectively), which have been implicated in TGN to plasma membrane traffic (Preuss *et al.*, 2006; Feraru *et al.*, 2012; Asaoka *et al.*, 2013; Qi and Zheng, 2013; Li *et al.*, 2017). In both normal and plasmolyzed cotyledon epidermal cells, FKD1–GFP co-localizes with the plasma membrane markers YFP–NPSN12 and YFP–PIP1;4, while in root cells, FKD1–GFP localization strongly overlaps the initial uptake of FM4-64 into the plasma membrane and also associates with plasma membrane-localized PtdIns(4)P and PtdIns(4,5)P_2_ markers UBQ10:CITRINE–2×PH(FAPP1) and UBQ10:CITRINE–2×PH(PLCD1).

SFC/VAN3 has been proposed to act during clathrin-mediated endocytosis (Naramoto *et al.*, 2011). However, only rare FKD1–GFP-marked vesicles co-localize with FM4-64 over the time course of labeling. Moreover, FKD1–GFP does not co-localize with YFP–RABF2b, a marker of both the EE (Ueda *et al.*, 2004) and late endosome (Lee *et al.*, 2004; Ebine *et al.*, 2011). We suggest that FKD1 is not primarily involved in endocytosis, and that the involvement of SFC in endocytosis may be restricted to the proportion that does not associate with FKD1. FKD1–GFP does co-localize with RABA1e, which has been described as a marker of early and recycling endosomal compartments, based on BFA sensitivity and partial association with FM4-64 (Geldner *et al.*, 2009). The co-localization of RabA1a, RabA1b, and RabA1c (Asaoka *et al.*, 2013), the secretory defects in quadruple mutants of RabA1a, RabA1b, RabA1c, and RabA1d (Asaoka *et al.*, 2013; Qi and Zheng, 2013), and the similar response to endosidin16 (ES16), which is thought to target secretion via RABA and RABE proteins (Li *et al.*, 2017), together with the high amino acid sequence identity (70–80%) amongst the seven RABA1p proteins suggest that they may act redundantly within the secretory pathway. While careful determination of the localization of RabA1e has not been carried out, we suggest that, like other members of the RabA1 group, it may be acting within the post-Golgi secretory pathway.

### FKD1 localization is correlated with the phosphoinositide profile

Both the SFC PH domain and the FKD1 PL domain have been suggested to bind to PtdIns(4)P (Carland and Nelson, 2009; Naramoto *et al.*, 2009). A previous study showed that either the FKD1 PL or the SFC PH domain was sufficient for localization of the SFC/FKD1 complex (Naramoto *et al.*, 2009) and our analysis further suggests that SFC may have a higher affinity for its docking sites than FKD1. In cells expressing only FKD1–GFP, FKD1–GFP localization was often cytosolic, whereas when SFC–YFP was expressed together with FKD1–GFP, FKD1–GFP was never cytosolic. This may suggest that SFC serves to recruit or increase the affinity of FKD1 for certain vesicles, as has been proposed for the increased localization to punctae of ENDOSOMAL RAB EFFECTOR WITH PX-DOMAIN (EREX) when expressed with its interacter, RAB7 (Sakurai *et al.*, 2016). Interestingly, the localization of FKD1–GFP changes during pavement cell development, with more localization in the punctae as lobes are forming (2.5 DAG), and more cytosolic localization both before and after lobe formation (2 DAG and 3 DAG). One possibility is that the endogenous levels of SFC affect FKD1 localization; a second, not necessarily mutually exclusive, possibility is that the phosphoinositide profile of cells changes during leaf development, as has been shown for root and floral development (Nakamura *et al.*, 2014; Rodriguez-Villalon *et al.*, 2015), and that FKD1 localization responds accordingly. Consistent with PtdIns(4)P being required for FKD1 membrane localization, 35S:FKD1–GFP was more frequently cytosolic in cotyledon pavement cells within the *cvp2cvl1* double mutant background. Surprisingly, although PI4Kβ1 and PI4Kβ2 are important for generation of PtdIns(4)P and polarized secretion during root hair expansion (Preuss *et al.*, 2006), 35S:FKD1–GFP localization in cotyledon epidermal cells is unchanged in the *pi4kβ1β2* double mutant. It is possible that other PI4K activities (Mueller-Roeber, 2002) act redundantly in these cells to generate PtdIns(4)P.

Whereas SFC becomes completely cytosolic in the *cvp2cvl1* double mutant background (Naramoto *et al.*, 2009), a small proportion of 35S:FKD1–GFP remains in the punctae. A possible explanation is that the FKD1 PL domain is more promiscuous than the PH domain of SFC/VAN3, and can interact with phosphoinositides other than PtdIns(4)P. While FKD1–GFP does not co-localize with the PtdIns(3)P marker UBQ10:CITRINE–2×FYVE(HRS), it co-localizes strongly with the PtdIns(4)P marker UBQ10:CITRINE–2×PH(FAPP1) as well as the PtdIns(4,5)P_2_ marker UBQ10:CITRINE–2×PH(PLCD1), both of which localize to the plasma membrane (Simon *et al.*, 2014; Tejos *et al.*, 2014). Interestingly, plants mutant for both *PI5K1* and *PI5K2*, which phosphorylate PtdIns(4)P to produce PtdIns(4,5)P_2_, lack proper PIN1 localization in developing veins, and develop disconnected veins in both cotyledons and leaves (Tejos *et al.*, 2014), a phenotype similar to *fkd1* and *sfc*. It is possible that one mechanism by which the phosphoinositides regulate PIN1 localization is through recruitment of FKD1 and SFC.

### FKD1 and SFC act in a BFA-insensitive pathway

Neither FKD1–GFP nor SFC–YFP punctae are affected by BFA treatment of root cells, indicating that FKD1 and SFC are localized to a BFA-resistant compartment within the secretory pathway. Interestingly, although SFC–YFP strongly co-localizes with RabA1c, a large proportion of RABA1c becomes associated with the BFA compartment, as has previously been demonstrated for RABA1b (Asaoka *et al.*, 2013). Moreover, as for RABA1b (Asaoka *et al.*, 2013), some RabA1c punctae do not become incorporated into the BFA compartment. The BFA-insensitive RabA1c punctae do not completely overlap with the SFC–YFP punctae, suggesting that the SFC–YFP- and RABA1c-labeled punctae are in partially distinct domains.

Both *fkd1* and *sfc* mutants (Scarpella *et al.*, 2006; Hou *et al.*, 2010) alter PIN1 localization within developing provascular cells. In particular, *fkd1* mutants lack apical localization of PIN1 within the bipolar cells and the ULD (Hou *et al.*, 2010). While the mechanism of basal PIN1 localization has been extensively studied and involves recycling via GNOM (Geldner *et al.*, 2003; Kleine-Vehn *et al.*, 2008*b*), its apical localization occurs in only a subset of PIN1-expressing cells, and is poorly understood. Interestingly, PIN1 becomes localized to the apical membrane of root cells following prolonged BFA exposure (Kleine-Vehn *et al.*, 2008*a*), suggesting that the apical targeting of PIN1, which is absent within the leaf provascular cells of *fkd1* mutants, is through a BFA-insensitive pathway. A recent study (Li *et al.*, 2017) supports the BFA insensitivity of apical and lateral trafficking, and further reveals that this trafficking is sensitive to the drug endosidin 16, which seems to act by targeting RABA- and RABE-GTPases. The strong co-localization of FKD1–GFP and SFC–YFP with RABA-GTPases, and the BFA resistance of both FKD1–GFP and SFC–YFP suggest that these proteins may be acting in provascular cells to direct apical trafficking of PIN1 from the TGN to the plasma membrane. Although the RABA-GTPases, FKD1, and SFC have all been implicated in PIN1 trafficking to the apical membrane, the different BFA sensitivity of the RABA compartment compared with the FKD1 and SFC compartment suggests that they must have distinct, although possibly overlapping, functions.

SFC is an ARF-GAP of the ACAP class, and is predicted to act upon members of the ARF1 group, represented by ARFa1a–ARFa1f in Arabidopsis. FKD1 co-localized moderately or strongly with all members of the ARF1 group tested (ARFA1a, ARFA1c, ARFA1d, and ARFA1e). ARF1 single mutants have no visible phenotype, suggesting that the high sequence similarity (>95%) reflects cellular redundancy (Xu and Scheres, 2005). However, a dominant-negative allele of ARF1A1c (*bex-1*) affects the exocytosis or recycling of PIN1 to the plasma membrane (Tanaka *et al.*, 2014). Our finding that FKD1 co-localizes with ARF1 proteins, and both FKD1 and SFC localize to a RABA secretory compartment, together with the mislocalization of PIN1 in *fkd1*, *sfc*, or *bex-1* mutants (Scarpella *et al.*, 2006; Hou *et al.*, 2010; Tanaka *et al.*, 2014) suggests that FKD1, SFC, and the ARFA1 proteins may be working together to traffic PIN1 properly to the plasma membrane.

The association of FKD1, SFC, and ARF1 proteins suggests that an as yet unidentified ARF-GEF could also be a part of this complex. Plants contain two ARF-GEF subfamilies (i) the GBF subfamily that includes GNOM, GNOM LIKE1 (GNL1), and GNL2; and (ii) the BIG subfamily, which includes five members (BIG1–BIG5) (Richter *et al.*, 2007). ARF1A1c co-localizes with a number of ARF-GEFs including GNOM, GNL1, BEN1/MIN7/BIG5 (Tanaka *et al.*, 2014), BIG3, and BIG4 (Richter *et al.*, 2014), and BIG3 has been shown to catalyze the nucleotide exchange of ARFA1c in a BFA-insensitive manner (Nielsen *et al.*, 2008). Thus, it seems that the ARF1 group might be quite promiscuous in its use of ARF-GEFs.

Members of the GBF and BIG families have been attributed roles in both endocytic and secretory pathways. GNOM and GNL1 have recently both been found to work in an early secretory pathway which is important for localization of newly synthesized PIN1 to the basal plasma membrane (Doyle *et al.*, 2015). The BIG family (BIG1–BIG4) proteins have been suggested to have a role in secretion of proteins, including newly synthesized PIN1 proteins, from the TGN to the plasma membrane (Richter *et al.*, 2014). BIG5/BEN1 (*BFAVISUALIZED ENDOCYTIC TRAFFICKING DEFECTIVE*)/MIN7 localizes to the TGN/EE and has been suggested to be involved in early endosomal trafficking events through the TGN, which is essential for polar localization of PIN1 and PIN2 proteins (Tanaka *et al.*, 2009, 2013). Also, *min7* mutants are defective in polarized callose deposition, suggesting a possible role for BIG5/BEN1/MIN7 in secretion (Nomura *et al.*, 2006).

While SFC/VAN3 has been proposed to interact with GNOM at the plasma membrane and be required for clathrin-mediated endocytosis (Naramoto *et al.*, 2011), it seems unlikely that FKD1, which shows only rare co-localization with FM4-64-labeled vesicles, would be part of this complex. Since both FKD1- and SFC-labeled compartments are BFA insensitive, we propose that they also partner with a BFA-resistant ARF-GEF. In Arabidopsis, out of the eight ARF-GEFs, only three (GNL1, BIG3, and BEN1/MIN7/BIG5) are resistant to BFA (Geldner *et al.*, 2003; Nielsen *et al.*, 2006; Richter *et al.*, 2014). Based on the localization of these ARF-GEFs, it seems that BIG3 or BIG5, which, like FKD1 and SFC localize to the TGN/EE, are more likely candidates than GNL1, which primarily localizes to the Golgi (Geldner *et al.*, 2003; Naramoto *et al.*, 2014).

## Supplementary data

Supplementary data are available at *JXB* online.

Table S1. Cell size and shape of 2.5 DAG cotyledon epidermal pavement cells of *fkd1* transformed with 35S:FKD1–GFP and *cvp2cvl1* into which 35S:FKD1–GFP has been introgressed.

Fig. S1. Introduction of 35S:FKD1–GFP into *fkd1* or 35S:SFC–YFP into *fkd2* results in the wild-type leaf vascular pattern.

Fig. S2. 35S:FKD1–GFP and 35S:SFC–YFP are insensitive to BFA treatment.

Fig. S3. 35S:FKD1–GFP localizes to the plasma membrane in both unplasmolyzed and plasmolyzed cells.

Fig. S4. Subcellular localization of 35S:FKD1–GFP with ARFA1 proteins.

## Supplementary Material

Supplementary Table S1 and Figures S1-S4

## Abbreviations:

ARF: ADP ribosylation factor
ARF-GAP: ADP ribosylation factor GTPase-activating protein
ARF-GEF: ADP ribosylation factor guanine nucleotide exchange factor
BFA: brefeldin A
CFP: cyan fluorescent protein
*CVL1*: *CVP2 LIKE1*
*CVP2*: *COTYLEDON VASCULAR PATTERN2*
DAG: days after germination
*FKD1*: *FORKED1*
GFP: green fluorescent protein
*GNL1*: *GNOM-LIKE1*
LLD: lower loop domain
PCC: Pearson’s coefficient of correlation
PH: Plekstrin homology
PL: Plekstrin like
*PIN1*: *PINFORMED1*
PIP1;4: , aquaporin
RAB: RAS gene from rat brain
RFP: red fluorescent protein
*SFC*: *SCARFACE*
*SYP61*: *SYNTAXIN OF PLANTS61*
TGN: *trans*-Golgi network
ULD: upper loop domain
*VAB*: *VAN3 BINDING PROTEIN*
*VAN3*: *Vascular Network Defective 3*
YFP: yellow fluorescent protein.
