# Supplementary material for: Localization of Arabidopsis FORKED1 to a RABA-positive compartment suggests a role in secretion
Source: J Exp Bot. 2017 Jun 1;68(13):3375–90. doi: 10.1093/jxb/erx180 (PMC5853234; doi:10.1093/jxb/erx180)
Supplement: Supplementary Table S1 and Figures S1-S4 [file erx180_suppl_supplementary_table_s1_figures_s1_s4.pdf]

Supplementary Table 1. Cell size and shape of 2.5 DAG cotyledon epidermal pavement cells *fkd1* transformed with 35S:FKD1-GFP and *cvp2cvl1* into which 35S:FKD1-GFP has been introgressed. Epidermal pavement cells (n = number of cells) were analysed for area (A) and perimeter (P) using NIH image and undulation index (UI) was calculated using the formula $UI=P/(2\pi\sqrt{(A/\pi)})$. Data was analysed for significant differences using a Student's t-test, and no differences were found.

	fkd1 (n=174)	*cvp2cvl1* (n=134)	P-value (Student's t-test)
area	450.8 ± 304.1	441.8 ± 248.7	0.78
perimeter	89.3 ± 40.4	90.7 ± 34.4	0.74
Undulation index	1.24 ± 0.12	1.24 ± 0.13	0.99

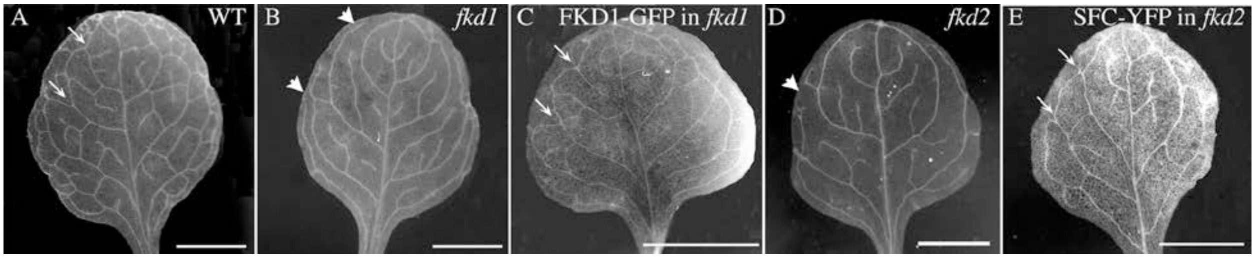

Supplementary Figure 1. Introduction of 35S:FKD1-GFP into *fkd1* or 35S:SFC-YFP into *fkd2* results in wild type leaf vascular pattern.

Vein pattern in cleared first leaves of wild type (A), *fkd1* (B), *fkd1* transformed with 35S:FKD1-GFP (C), *fkd2* (D) and *fkd2* transformed with 35S:SFC-YFP. Arrow in A, C and E indicate junctions between secondary veins; arrowheads in B and D indicate open secondary veins. Scale bar = 2 mm.

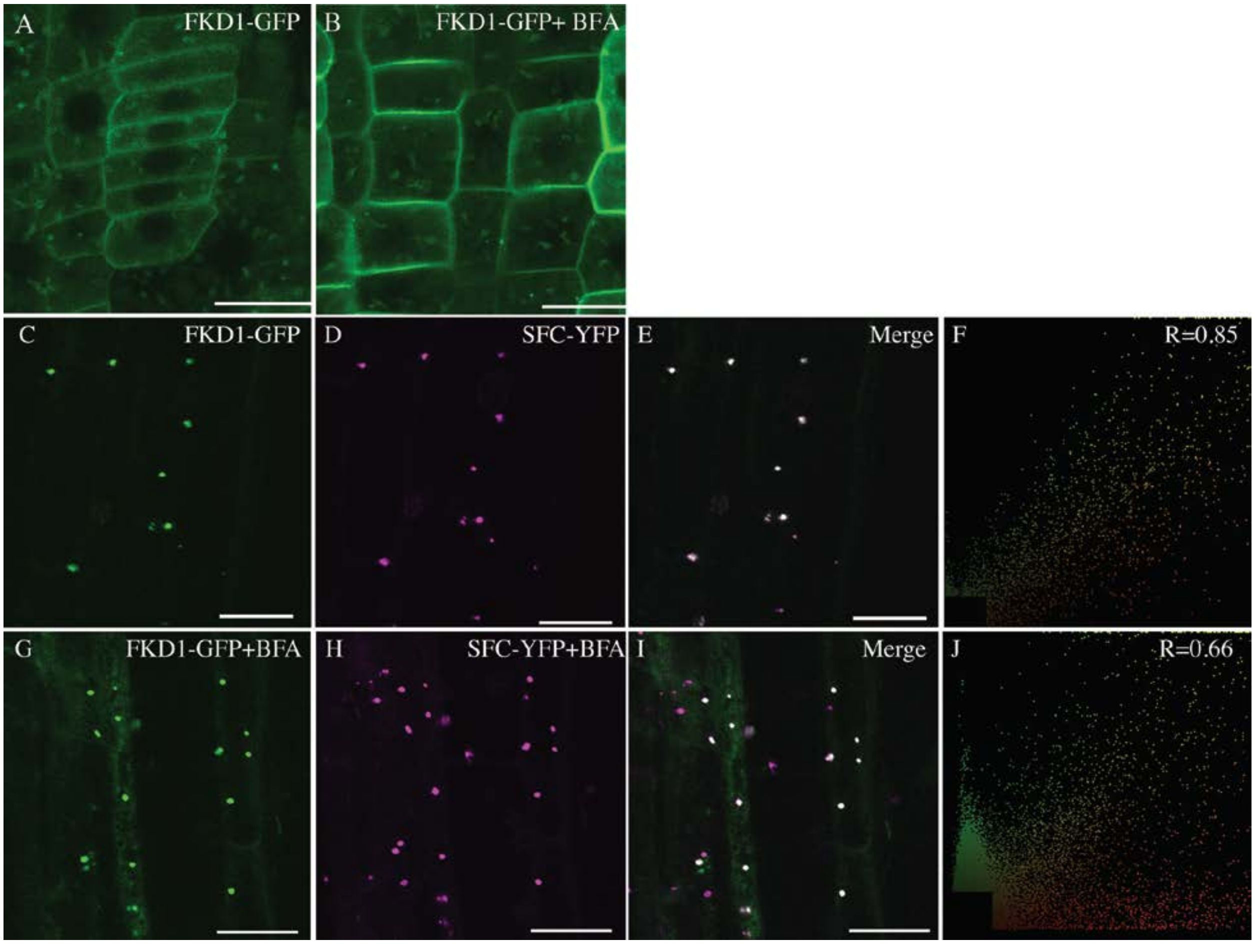

Supplementary Figure 2: 35S:FKD1-GFP and 35S:SFC-YFP are insensitive to BFA treatment

Localization of 35S:FKD1-GFP with DMSO (A) or with 50 μM BFA in DMSO (B) in root cells of stably transformed Arabidopsis seedlings at 2.5DAG. Co-localization of FKD1-GFP with 35S:SFC-YFP following DMSO (C-F) or 50 μM BFA in DMSO (G-J) in root cells of stably transformed Arabidopsis seedlings at 2.5DAG. For the co-localization, C and G are 35S:FKD1-GFP alone; D and H are 35S:SFC-YFP alone; E and I are the merged images; F and J are scatter plots of the merged image with Pearson's coefficient of Correlation (R) values. (Scale bar:10μm).

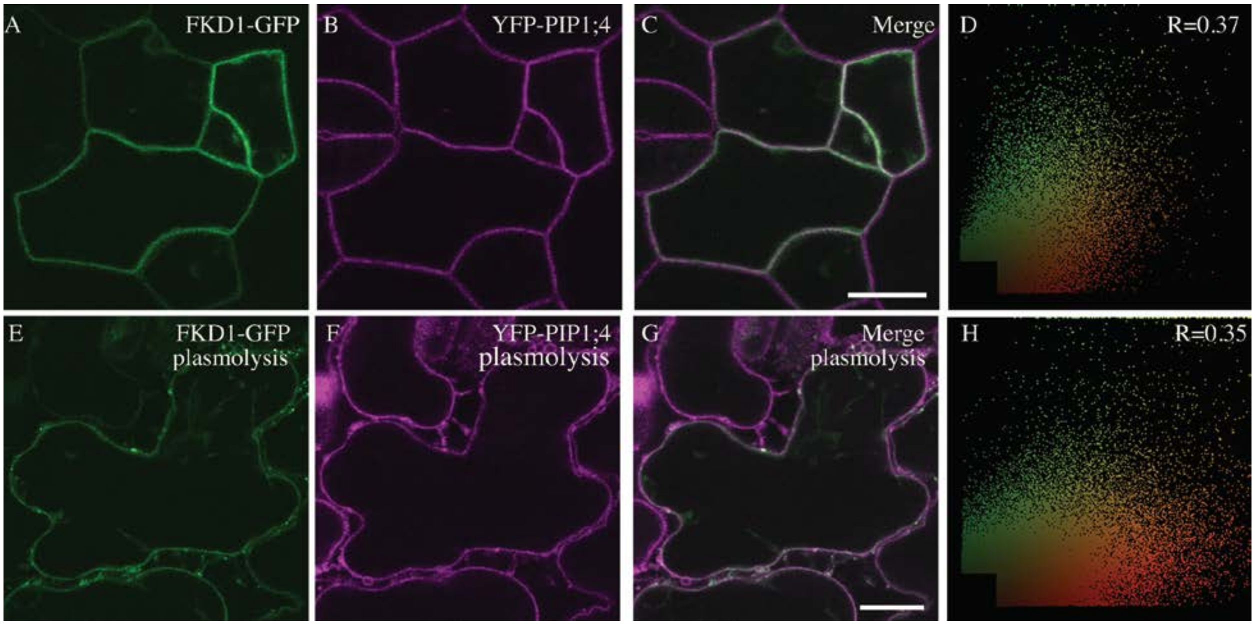

Supplementary Figure 3. 35S:FKD1-GFP localizes to the plasma membrane in both unplasmolyzed and plasmolyzed cells.

Localization in stably transformed 2.5 DAG Arabidopsis cotyledon pavement cells of 35S:FKD1-GFP with plasma membrane marker UBQ10:YFP-PIP1:4 (A-H) before (A-D) and after (E-G) plasmolysis treatment (800 mM mannitol, 3 hours). A and E are 35S:FKD1-GFP alone; B and F are UBQ10:YFP- PIP1:4: C and G are the merged image; D and H are scatter plots of the merged image with Pearson's coefficient of Correlation (R) values. (Scale bar: 10μm).

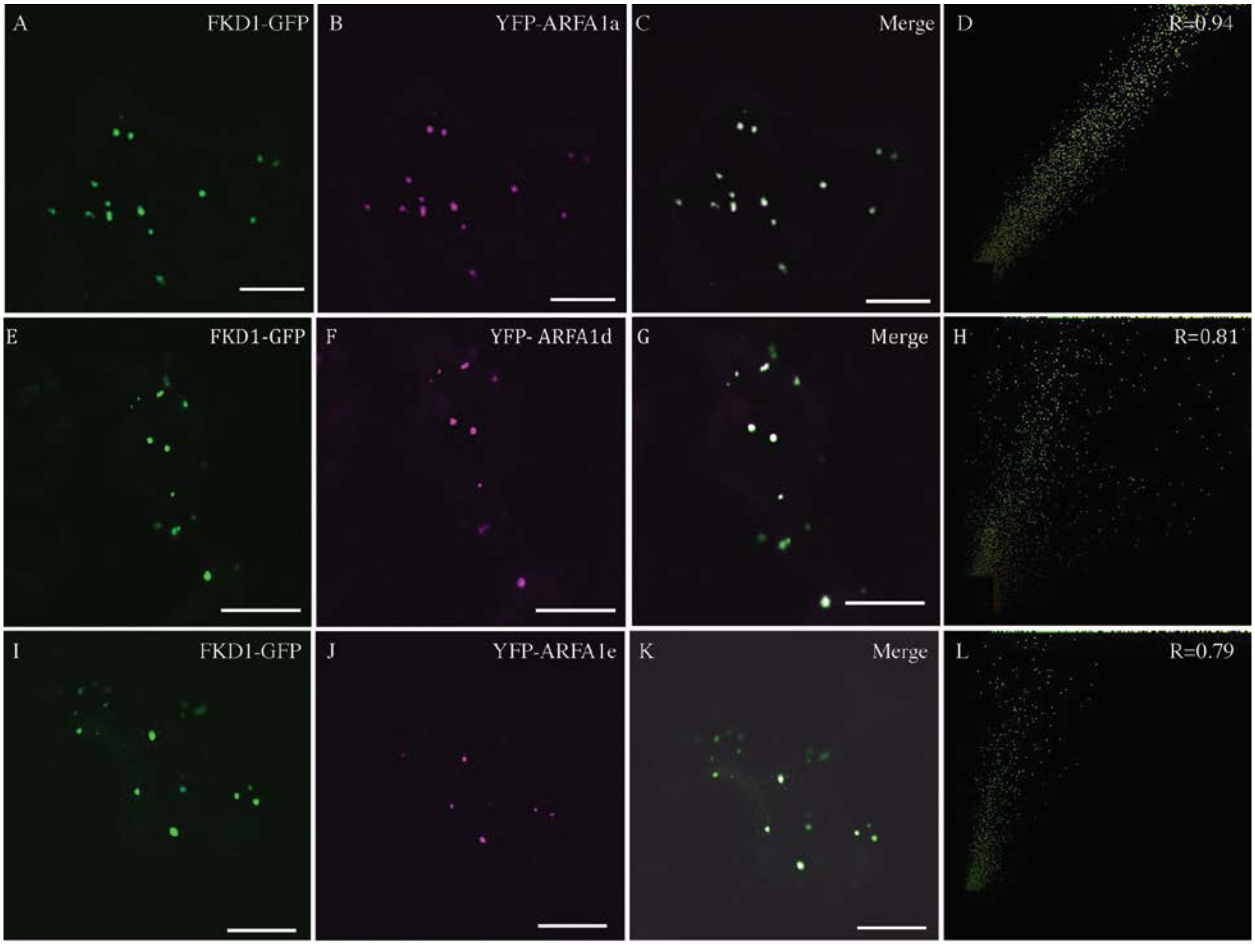

Supplemental Figure 4: Subcellular localization of 35S:FKD1-GFP with ARFA1 proteins

35S:FKD1-GFP with ARFA1a (A-D), ARFA1d (E-H), ARFA1e (I-K) transiently expressed in Nicotiana. A, E and I are FKD1 alone; B, F and J are ARFA1a, ARFA1d and ARFA1e fused to YFP. C, G and K are the merged images. D, H and L are scatter plots of the merged image with Pearson's coefficient of Correlation (R) values. (Scale bar:10µm).
